# Impact of elevated maternal HIV viral load at delivery on T-cell populations in HIV exposed uninfected infants in Mozambique

**DOI:** 10.1186/s12879-015-0766-6

**Published:** 2015-02-03

**Authors:** Nilsa de Deus, Cinta Moraleda, Celia Serna-Bolea, Montse Renom, Clara Menendez, Denise Naniche

**Affiliations:** National Institute of Health, Maputo, Mozambique; Manhiça Health Research Centre (CISM), Manhiça, Mozambique; Barcelona Centre for International Health Research (CRESIB), Hospital Clinic, Universitat de Barcelona, C/Rossello 132, 4°, Barcelona, Spain

**Keywords:** HIV-exposed uninfected, T-cell, Naïve, Memory, Maternal HIV-RNA load, Africa

## Abstract

**Background:**

HIV-uninfected infants born to HIV-infected mothers (HIV-exposed uninfected, HEU) have been described to have immune alterations as compared to unexposed infants. This study sought to characterize T-cell populations after birth in HEU infants and unexposed infants living in a semirural area in southern Mozambique.

**Methods:**

Between August 2008 and June 2009 mother-infant pairs were enrolled at the Manhiça District Hospital at delivery into a prospective observational analysis of immunological and health outcomes in HEU infants. Infants were invited to return at one month of age for a clinical examination, HIV DNA-PCR, and immunophenotypic analyses. The primary analysis sought to assess immunological differences between HEU and unexposed groups, whereas the secondary analysis assessed the impact of maternal HIV RNA viral load in the HEU group. Infants who had a positive HIV DNA-PCR test were not included in the analysis.

**Results:**

At one month of age, the 74 HEU and the 56 unexposed infants had similar median levels of naïve, memory and activated CD8 and CD4 T-cells. Infant naïve and activated CD8 T-cells were found to be associated with maternal HIV-RNA load at delivery. HEU infants born to women with HIV-RNA loads above 5 log_10_ copies/mL had lower median levels of naïve CD8 T-cells (p = 0.04), and higher median levels of memory CD8 T-cells, (p = 0.014).

**Conclusions:**

This study suggests that exposure to elevated maternal HIV-RNA puts the infant at higher risk of having early T-cell abnormalities. Improving prophylaxis of mother to child HIV programs such that more women have undetectable viral load is crucial to decrease vertical transmission of HIV, but may also be important to reduce the consequences of HIV virus exposure in HEU infants.

## Background

Mother to child transmission (MTCT) of HIV occurs at a rate of 15% to 40% in a breastfeeding population in the absence of prevention measures [1]. However, prevention of MTCT of HIV (pMTCT-HIV) through antiretroviral prophylaxis, cesarean deliveries and formula feeding has successfully reduced MTCT to less than 1% in resource-rich countries [2-4]. In Sub-Saharan African countries, the last decade has witnessed a major rollout of pMTCT-HIV programs, which has contributed to dropping rates of vertical transmission of HIV. However, with antenatal clinic HIV prevalence reaching 30% in some countries [5,6], many infants are potentially exposed to HIV, although remaining uninfected. These HIV-exposed uninfected (HEU) infants may have immunological alterations which could lead to weakened responses to infections and vaccines and vulnerability to disease [7].

There is clear evidence that HEU infants present cytopenias including neutropenia as well as imbalances in levels of total CD4 and CD8 T-cells [8-20]. There is also increasing evidence that other immune abnormalities may be present in HEU infants, albeit to differing degrees, such as, altered cytokine production or memory/naïve T-cell skewing [10,11,13-15,21-24]. The duration of these abnormalities is not clear. HEU infants may also have lower thymus output and increased immune activation as compared to unexposed infants [9,14,21]. However, reduced thymic size in HEU was not found to lead to differences in T cell phenotypes or function between HEU and unexposed infants at 15 months of age [25]. Some studies have suggested an important role of maternal and infant antiretroviral exposure in triggering immunological alterations, but the impact remains unclear [22,26]. HEU studies on lymphocytes and thymus output have mainly been conducted in Europe, North America and Brazil. The situation may be more complex in Sub-Saharan Africa [19] where independently of HIV, Africans have been shown to have lower levels of CD4, naïve T-cells and increased levels of activated T-cells as compared to Europeans [27-31]. In addition, environmental and setting-specific factors affecting immunological indicators may vary widely in Sub-Saharan Africa. For example, in areas of high malaria endemicity such as Mozambique, cord blood lymphocytes from mothers infected with malaria are often primed to parasite antigens and exhibit higher level of activation [32,33].

The clinical consequences of immune alterations observed in HEU infants are unknown. There has been an observation that HEU infants may mount a weaker immune response to BCG than infants born to HIV-uninfected women [34,35]. Another consequence of impaired immunity could be that the severity, frequency and mortality associated with infections is exacerbated in HEU infants as compared to unexposed infants [36-39]; however it is not clear whether this is due to specific immune alterations [40-43] or to other mechanisms.

This study sought to characterize T-cell populations after birth in HEU infants and unexposed infants living in a semirural area in southern Mozambique where HIV clade C is the predominant circulating virus.

## Methods

### Study population

This study was conducted from August 2008 to August 2010 at the Manhiça District Hospital in southern Mozambique. The present study is integrated into a prospective observational analysis of immunological and health outcomes in infants born to HIV-infected mothers which has been described elsewhere [20]. National guidelines for pMTCT-HIV during the study followed the 2006 World Health Organization (WHO) recommendation [44]. At the time of the study, Highly Active Antiretroviral Therapy (HAART) was indicated for women in WHO disease stage 3 or 4 or CD4 < 250 counts/μl. For infants whose mothers received a complete pMTCT-HIV was based on a single dose of nevirapine (NVP) and daily zidovudine (AZT) during 1 week post-delivery and for those whose mothers did not receive a complete prophylaxis a single dose of NVP and 4 weeks of daily AZT was recommended. After the first month of the study, the pMTCT-HIV national recommendation for children changed, and 4 weeks of daily AZT was recommended for all infants born to HIV-infected mothers. pMTCT-HIV was considered complete for the mother if she had received all treatments according to the guidelines, and for the newborn if he/she had received one dose of nevirapine in the first 72 hours after birth plus daily zidovudine for 4 weeks after delivery [44]. Women were counselled to exclusively breastfeed through 6 months. All HIV-infected mothers and HIV-infected infants were referred to the day hospital for clinical management according to national guidelines.

All participating mothers gave written informed consent, and the study protocol was approved by the Mozambican National Bioethics and the Hospital Clinic of Barcelona Ethics Review Committees.

### Study procedures

At enrolment HIV serology testing was performed in the mothers using the Determine HIV-1/2 Rapid Test (Abbott Laboratories, Abbott Park, IL) and positive results were confirmed by the Uni-Gold Rapid Test (Trinity Biotech Co., Wicklow, Ireland) according to national guidelines. A 5 mL venous sample was drawn from HIV-positive mothers for CD4 cell counts and viral load determinations. Infants were invited to return at one month of age for a clinical examination, HIV DNA-PCR, and blood drawing. Infants who had a positive HIV DNA-PCR test were not included in the analysis. Due to the difficult blood extraction, several children did not have enough blood for all the determinations. Only the children with all the parameters available were included in this analysis. The mother-infant pairs included: 1) HIV-infected mothers and their HEU infants and 2) HIV-uninfected mothers and their unexposed infants.

### HIV serological and virological determinations

Infants were tested for HIV-1 using the Amplicor DNA-PCR kit (Roche Diagnostics, Basel, Switzerland). HIV-RNA quantification was performed from cryopreserved plasma samples with the commercial Roche Amplicor Monitor, version 1.5 (Roche Diagnostics, Basel, Switzerland). The assay had a lower limit of detection of 400 HIV-1 RNA copies/mL. For the purpose of analyses, plasma HIV-1 RNA concentrations below the limit of detection were assigned the value of 200 copies/mL.

### Immunophenotypic analysis

CD4 and CD8 counting was performed from fresh whole blood after staining with labelled antibodies: CD4, CD3, CD8, and CD45 in TruCount tubes (Becton Dickinson Biosciences, California, USA). Samples were assessed by flow cytometry on a FACS Calibur (Becton Dickinson). Analysis was completed using Multiset software (Becton Dickinson Biosciences, California, USA).

To assess the percentage of activated CD4 and CD8 T lymphocytes, cell staining was performed with CD3, CD4 or CD8, CD38, and HLA-DR antibodies. Activated T-cells were defined as those CD4 or CD8 T-cells expressing both CD38 and HLA-DR surface markers. The percentage of naïve CD4 and CD8 was determined with CD62L and CD45RA antibodies whereas the percentage of memory CD4 and CD8 T-cells was evaluated by pan-CD45RO antibody staining.

### Statistical analysis and definitions

HEU infants were defined as those having a negative HIV-DNA PCR test at 1 month of age. Parity was categorized as primiparous if this was the first pregnancy and multiparous if the mother had had any previous pregnancy. Low birth weight was defined as birth weight < 2500 grams.

Comparisons between groups for proportions were assessed using the chi-square test or Fisher’s exact test where appropriate. For the T-cell population studies, analyses were made on continuous variables. The Kruskal-Wallis test was used to compare independent continuous variables between groups. The Wilcoxon signed rank test was used for analysis of paired data. The Spearman correlation was used to assess correlations between non-normally distributed continuous variables.

Statistical analysis was performed using STATA version 12.0 (StataCorp, College Station, TX). The primary analysis sought to assess immunological differences between HEU and unexposed groups, whereas the secondary analysis assessed the impact of the maternal HIV RNA viral load within the HEU group.

## Results

### Study population

Between August 2008 and June 2009, 318 mothers, 158 HIV-infected women and 160 HIV-uninfected women were enrolled at the Manhiça District Hospital [20]. Of these, 110 HIV-infected and 75 HIV-uninfected women came to the clinic at the infant’s one month visit and were included in this study. Ten infants were determined to be HIV-infected at 1 month of age and excluded from the analysis. Of the remaining 175 mother-infant pairs, 130 infants had all immunological measures at 1 month of age, (74 infants born to HIV-infected mothers and 56 infants born to HIV-uninfected mothers). The 45 mother-infant pairs lost to follow up were comparable to the 130 used in this analysis in terms of demographic characteristics (data not shown).

Demographic and clinical characteristics of mothers and their infants at delivery were comparable between HEU and unexposed groups (Table 1). The 130 mother-infant pairs included in this analysis were comparable to the overall study population in terms of parity, age and birth weight and for HIV-infected women in terms of CD4 counts and HIV RNA levels (data not shown) [20]. At delivery the median maternal CD4 T-cell counts of HIV-infected mothers was 624 cells/μl (IQR: 495–837) and 17% of the women had levels below 350 CD4 cells/μl. Only 20.63% of the HIV-infected women had undetectable HIV RNA at delivery and median HIV RNA load was 3.91 log_10_ copies/mL (IQR: 2.97-4.67). Eighty-two percent of HIV-infected mothers received complete pMTCT-HIV. At one-month of age, close to 90% of infants were receiving exclusive breastfeeding.

**Table 1:** **Demographic and clinical characteristics of mothers and their infants at delivery according to maternal HIV serostatus (n = 130)**

	**HIV-infected**		**HIV-uninfected**		**P**
	**N = 74**		**N = 56**		
Parity (n, %)					
primigravidae	14	18.92	11	19.64	0.917
multigravidae	60	81.08	45	80.36	
Age (yrs) [median (IQR)]		25 (22–30)		23 (20–31)	0.335
Low Birth weight, (n, %)	3	4.17	1	1.82	0.453
Birth weight (gr) [median (IQR)]		3000 (2800–3300)		3100 (2900–3350)	0.253
Sex, (n, %)					
male	42	56.76	23	41.07	0.077
female	32	43.24	33	58.03	
Maternal HIV RNA log_10_* (copies/mL) [median (IQR)]		3.91 (2.97-4.67)		NA	
Maternal CD4 counts (cells/μl) [median (IQR)]		624 (495–837)		NA	

IQR: interquartile range.

*Analysis is based on n = 63 infants with maternal HIV RNA measures available.

### Distribution of T-cell populations among HEU and unexposed infants at 1 month of age

To investigate the impact of maternal HIV infection on the distribution of infant T-cells subsets, percent activated, naïve and memory CD4 and CD8 T-cell subsets were assessed in HEU and UE infants.

**Figure 1:**
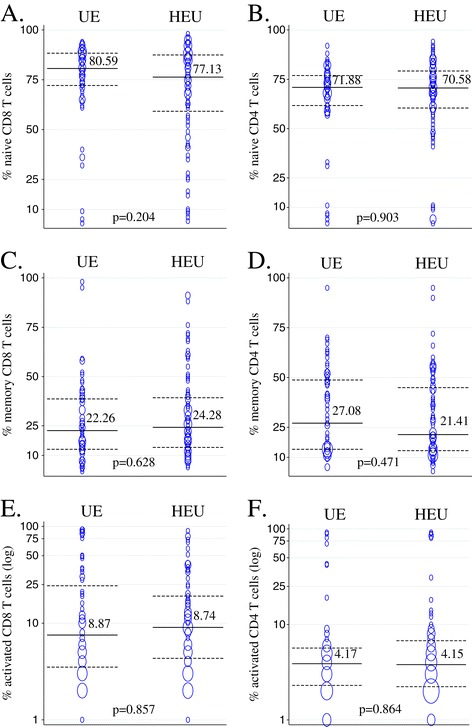
**Distribution of T-cell populations among HIV-exposed infected (HEU) and unexposed (UE) infants at 1 month of age.** Medians for data from HEU (n = 74) and UE (n = 56) are indicated and plotted in solid lines with dotted lines representing interquartile ranges. The size of bubbles is proportional to number of observations. **A**, **C**, and **E** show naïve, memory and activated CD8 T-cell populations and **B**, **D** and **F** show naïve, memory and activated CD4 T-cell populations.

At one month of age, the seventy-four HEU and the fifty-six unexposed infants had similar median levels of naïve, memory and activated CD8 and CD4 T-cells (Figure 1).

### The impact of maternal HIV viral load on levels of naïve and memory CD8 T-cells

**Figure 2:**
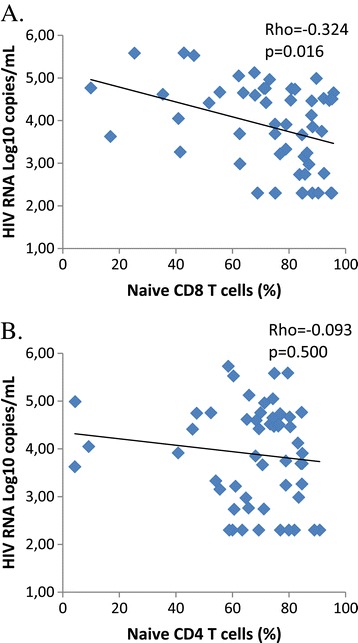
**Correlation between maternal HIV RNA viral load at delivery and infant level of naïve CD8 T cells (A) and naïve CD4 T cells (B) at one month of age.** Spearman correlation coefficient Rho and its corresponding p-value is indicated for each graph.

Within HEU infants, levels of naïve CD8 T-cells showed a significant negative association with maternal HIV RNA viral load (Spearman correlation: rho = −0.324, p = 0.016) whereas levels of naïve CD4 T-cells were not significantly associated with maternal HIV RNA (Figure 2). Memory CD8 T-cells and activated CD8 T-cells were positively associated with maternal HIV RNA viral load (memory: Spearman rho = 0.293 p = 0.031 and activated: Spearman rho = 0.312 p = 0.02). Percentage of activated and memory CD4 T-cells were not significantly associated with maternal HIV RNA viral load (p = 0.180, p = 0.733 respectively).

When HEU infants were categorized according to their mothers HIV RNA at delivery, HEU infants born to women with HIV viral load above 5 log_10_ copies/mL had significantly lower levels of naïve CD8 T-cells and higher levels of memory CD8 T-cells at 1 month of age as compared to infants born to women with HIV RNA level below 5 log_10_ copies/mL (Table 2). Maternal HIV RNA above 5 log_10_ copies/mL was also associated with increased levels of activated CD8 T-cells although this did not reach significance. There were no associations between maternal HIV RNA loads and levels of infant naïve, activated or memory CD4 T-cells (Table 2). The infants born to women with high HIV RNA levels remained HIV-uninfected at subsequent visits although two were lost to follow up at the 12 month visit within the prospective observational study.

**Table 2:** **Levels of T-cell populations in HIV exposed uninfected infants at 1 month of age according to maternal HIV RNA viral load at delivery**

**Cell population**	**HIV RNA ≥ 5 log**_**10**_ **(n = 6) [median (IQR)]**	**HIV RNA < 5 log**_**10**_ **(n = 57) [median (IQR)]**	p-value*
**CD8 T-cells**			
counts (cells/μl)	1585 (1080–1702)	1112 (71–1447)	0.14
activated (%)	25.8 (11.2-40.3)	8.8 (3.3-18.3)	0.09
naive (%)	54.3 (42.8-67.8)	79.8 (67.6-88.2)	0.04
memory (%)	54.6 (30.5-75.8)	21.9 (14.3-40.3)	0.014
**CD4 T-cells**			
counts (cells/μl)	2594 (2174–3185)	2291 (1955–3028)	0.337
activated (%)	5.3 (4.0-7.8)	3.5 (2.1-6.5)	0.557
naive (%)	70.1 (60.4-74.8)	70.3 (60.6-78.9)	0.79
memory (%)	19.8 (15.4-21.7)	23.9 (13.2-45.8)	0.574

Analysis is based on n = 63 infants with maternal HIV RNA measures available.

*P-value from kruskal-wallis test.

Higher maternal CD4 counts at delivery were significantly associated with higher level of infant naive CD4 T-cells (spearman correlation: rho = 0.365 p = 0.0018) but not with naïve CD8 T-cells (spearman correlation: rho = 0.007 p = 0.95) nor with memory CD4 or CD8 T-cells (p = 0.104 and p = 0.811 respectively).

## Discussion

In this study, at one month of age, HEU and unexposed infants had comparable median percentages of naïve, memory and activated CD4 T-cells and CD8 T-cells. However, within the HEU infant group, HEU born to women with high maternal HIV RNA level at delivery showed skewed naïve and activated CD8 T-cell populations at one month of age as compared to HEU infants born to women with lower viral load. In addition, maternal CD4 counts at delivery were positively associated with levels of infant naïve CD4 T-cells.

A naïve immune system with little previous antigen stimulation is expected to have high levels of naïve T-cells and low activation. With age, the levels of naïve cells decrease and memory cells increase reflecting antigen exposure [45,46]. HIV-infected infants show an activated phenotype whereby they display a decrease in naïve CD4 and/or CD8 T-cells and an increase in activated and memory T-cells [47]. Some studies have suggested that HEU infants also have naïve/memory T-cell skewing, albeit to a lesser extent, whereas other studies have not observed this skewing in HEU [11,13-15,19]. In the current study, differences in naïve/memory T-cell skewing were not observed at one month of age between HEU and UE. However, significantly lower median %CD4 T-cells and higher %CD8 T-cells were observed in these HEU as compared to UE [20]. The inconsistency in the literature regarding naïve/memory T-cell skewing in HEU infants leads to speculation as to the impact of factors such as time point of measurement, comparability of birth outcomes between HEU and UE infants, overall disease burden and maternal viral load. Studies of thymic naïve T-cell output in HEU infants by Nielsen et al., [14] found birth outcomes such as gestational age, birth weight and mode of delivery to impact observed infant naïve T cell levels. This could lead to confounding when assessing lymphocyte subset differences between HEU and UE which have very different birth outcomes. Additionally, time points for assessment of lymphocyte subsets have ranged from birth to adolescence, thus contributing to non-comparability of results. In our cohort, the birth weight, mode of delivery and feeding was not significantly different between HEU and unexposed infants. Since those infants requiring urgent care at birth were not included in our study, the absence of naïve/memory lymphocyte skewing in HEU at one month of age could be due to exclusion of those infants with poor birth outcomes. Alternatively, Manhiça is an endemic region of *P. falciparum* malaria and thus heightened malaria-induced immune activation in newborns could have masked differences due to HIV exposure. Our results showing correlation between maternal HIV viral load and infant CD8 T cell skewing suggest that only a sub population of HEU show T-cell skewing.

This study is one of the first to our knowledge to describe a relationship between maternal HIV RNA level and naïve/memory skewing in CD8 T-cell populations in HEU infants. This complements a recent study from Canada which showed that high maternal viral load was associated with higher levels of CD19+ B-cells in HEU infants [48]. Both studies point to a dose-dependent association of maternal HIV RNA level with lymphocyte subset skewing. In most cohorts studied in resource-rich countries, HIV viral load is undetectable or low due to effective pMTCT-HIV. In the current study, despite the existence of a pMTCT-HIV program, nearly 80% of women had detectable viral load at delivery. Among the HEU infants, linear associations were observed between levels of maternal HIV RNA viral load at delivery and naïve, pan-memory and activated CD8 T-cells. Maternal HIV viral load greater than 5 log_10_ copies/mL was associated with a significantly lower percentage of naïve CD8 T-cells and a higher percentage of memory CD8 T-cells as compared to HEU infants born to mothers with maternal HIV RNA below 5 log_10_ copies/mL. *In utero* infant exposure to maternal HIV particles and/or proteins could lead to lymphocyte subset skewing but may require a certain threshold or duration of maternal HIV exposure to be observed. The existence of such a threshold may help to explain inconsistent results in HEU immune abnormalities across studies. Additionally, in breastfeeding populations, the infant is potentially continuously exposed to HIV through breastmilk which would be higher in those women with higher plasma HIV RNA levels.

The main study limitations are the small sample size, particularly in the high maternal HIV RNA group, and the lack of evaluation of the duration of the T-cell skewing. Further studies including monitoring breast milk viral load exposure will be necessary. The analysis of memory T-cells was performed using the CD45RO marker, which is a non-specific marker of mature phenotype T-cells and does not distinguish between central and effector memory. The use of CD45RO may have underestimated levels of memory cells by excluding stem cell memory cells which are CD45RO [49] but this would have affected both HEU and UE groups. Other more specific markers that require greater than 4-color flow cytometry could not be assessed in Manhiça.

## Conclusions

In conclusion, HEU infants born to women with high maternal HIV RNA level at delivery had skewed naïve and activated CD8 T-cell populations at one month of age. Improving pMTCT-HIV programs such that more women have undetectable viral load is crucial to decrease vertical transmission of HIV, but may also be important to reduce the consequences of HIV virus exposure in HEU infants. The current recommendation of HAART for all HIV-infected pregnant women (the B+ option) is promising and will serve to reduce infant HIV exposure in Sub-Saharan Africa [50]. However, there will be a lag time until infant HIV exposure is eliminated in the region, thus elucidation of the clinical relevance of infant’s exposure to maternal HIV [20,51-53] and its impact on altered T-cell populations should remain a priority.

## Abbreviations

AZT: Zidovudine
HAART: Highly active antiretroviral therapy
HEU: HIV-exposed uninfected
MTCT: Mother to child transmission
NVP: Nevirapine
pMTCT-HIV: Prevention of MTCT of HIV
WHO: World Health Organization
